# Measuring the impact of participatory research in psychiatry: How the search for epistemic justifications obscures ethical considerations

**DOI:** 10.1111/hex.12988

**Published:** 2019-12-18

**Authors:** Phoebe Friesen, Sapfo Lignou, Mark Sheehan, Ilina Singh

**Affiliations:** ^1^ Department of Population Health Ethox Centre University of Oxford Oxford UK; ^2^ NEUROSEC Department of Psychiatry University of Oxford Oxford UK

**Keywords:** impact, mental health, participatory research, public and patient involvement, service users, survivor research

## Abstract

**Context:**

Both within politics and practice, the field of psychiatry is undergoing a significant transformation, as increasing emphasis is placed on the importance of involving those with lived experience in research. In response to this participatory turn, a push towards measuring the impact of patient involvement is also growing, seeking to identify how participation can improve research.

**Objective:**

This paper examines the recent push towards measuring impact in relation to justifications underlying the democratization of research in psychiatry, revealing a disconnect between the two, and harms that could result from a singular focus on measuring impact.

**Discussion:**

While those promoting and regulating participatory research tend to focus on the epistemic benefits of such research, many have pointed to both epistemic and ethical justifications underlying participatory research. The ethical reasons for involving service users loom especially large in psychiatry, given its unique history of abuse, the ways diagnoses can be utilized as tools for oppression, and the prevalence of coercion. The current focus on measuring the impact of involvement can be harmful, in that it obscures ethical reasons in favour of epistemic ones, potentially exacerbating issues common to participatory research, such as role confusion and ineffective, tokenistic participatory efforts.

**Conclusions:**

We argue that to take the ethical reasons behind involvement in mental health research seriously will involve looking beyond impact and towards sharing power. We suggest three ways this can be done: measuring more than impact, building service user capacities and sharing power in realms outside of research.

## 1 INTRODUCTION

### 1.1 The turn to participatory research

The field of psychiatry is currently undergoing a significant transformation. Both within politics and practice, increasing emphasis is being placed on the importance of involving those with lived experience in psychiatric research. As part of a wider movement towards the democratization of knowledge production in the health sciences, more and more funders are requiring research protocols to include plans to involve or engage patients or the public in the development, execution and dissemination of their findings.* Variously known as participatory research, user involvement (UI), public and patient involvement and engagement (PPIE), patient and service user engagement, co‐production, participatory action research (PAR),† user‐led research and citizen science, these new forms of investigation suggest that research is no longer solely the domain of the academic.‡ While some of these terms, like PPIE, refer to both public and patient involvement in research, our focus here is on the involvement of patients or service users, not lay people in general.§

In psychiatry, participatory research related to mental health is both more advanced than most other medical specialities and has a longer history.^7^ Patient groups, the recovery movement, consumer/ survivor/ ex‐patient (c/s/x/) communities and those invested in mad pride and mad studies have long pushed for the rights of service users to be involved in research that concerns them. In practice, as well as in mental health research, the involvement of service users is becoming more and more common. The workforce of peer specialists is growing steadily, and evidence for the efficacy of support offered by those with lived experience of being on the other side of the mental health system is increasing.^8^

### 1.2 Responses to the participatory turn

Both in the larger context of participatory health research in general, and in relation to participatory health research in psychiatry, responses from various stakeholders (eg researchers, patients, funders) have been mixed. For some, requirements to involve patients in research have appeared quite suddenly, with little justification or instruction, leading to role confusion and tokenism.^9^ Others have met the shift with skepticism, suggesting that it is a form of liberalism gone awry and is only making research more laborious and expensive.^10^ For others, the shift towards participation is seen as a welcome and long‐awaited development, signalling progress resulting from many years of activism and demands to change the structures of authority in knowledge projects.^11^ Still, others argue that the shift has not gone far enough and exemplifies yet another case of researchers extracting and exploiting the hard‐won knowledge of service user communities without giving due credit or making real change.^12^

In this paper, we critically examine an initiative that aligns with many of these responses: measuring the ‘impact’ of participatory research. Funding entities and universities that support and/or mandate PPI in research have been keen to measure impact, so as to provide stronger justification for the PPI endeavour and to discover ways to improve it. Particular emphasis has been placed on developing processes to measure how research processes and outputs are affected by participatory techniques. While this is a laudable aim, we argue that a focus on impact fails to acknowledge the diverse justifications underlying the participatory turn. Narrowing in on the case of mental health research, we make the case below that the push towards measuring the impact of participatory research can obscure the ethical justifications underlying such research, leading to a limited understanding of why inclusion matters, and direct harms to participatory practices. In the following section, we briefly outline the push towards measuring impact that has developed in response to the participatory turn. Second, we examine justifications that have been offered for the democratization of psychiatric research, resting on both epistemic and ethical grounds. Next, we argue that a focus on impact can direct attention away from the ethical reasons we ought to engage in participatory research in psychiatry, leading to several harms. Finally, we offer suggestions for how this focus can be broadened, and the ethical reasons underlying participatory mental health research can be taken into account. Through this analysis, we hope to make a contribution to important on‐going discussions about the purpose of patient involvement in mental health research and how such research should be evaluated.

## 2 MEASURING IMPACT

### 2.1 Measuring impact in participatory research

Predictably, a push towards evaluating participatory research has followed quickly on the heels of the participatory movement. Many are united under the goal of measuring the fruits of the participatory turn; those hoping to justify the democratization of research wish to see the proof in the pudding, those with doubts are eager to see their skepticism confirmed, and those who wish to understand the mechanisms that make participatory research most effective are also intrigued. This alignment has led to a burgeoning research program which seeks to measure the ‘impact’ of participatory research, focusing primarily on the ways in which participation leads to better research.

Recent systematic reviews of impact emphasize the ways in which participatory research can improve the process and outputs of research, by supporting the recruitment process, the dissemination of results or making research more relevant to users (eg through the development of more accessible materials, more appropriate outcome measures).^9^, ^13^ A recent review of the impact of participatory research by PCORI (the Patient‐Centered Outcomes Research Institute) summarized the ways in which engagement impacted the design, conduct and dissemination of research.^14^ Taking a slightly wider approach, INVOLVE's impact review examined how participatory research changed the agenda, ethics, design, delivery and implementation of research, as well as how it impacted stakeholders including the public, researchers, participants, organizations and the wider community.^15^ A review focusing solely on stakeholders, as opposed to the research process, reported on how researchers ‘developed a greater understanding and insight into their research area’ and the community ‘became more aware and knowledgeable about their condition’.^16^ ¶

### 2.2 Measuring impact in participatory research in psychiatry

In psychiatry, specifically, many are calling for evaluations of the impact of participatory research.^18^ Perhaps because of the politicized nature of many communities of mental health activists who wish to challenge the status quo, it has been reported that ‘there is a view that PPI [public and patient involvement] in research, particularly in mental health, is biased, anecdotal and carried out by people who are over‐involved’.^7^ This has contributed to a desire to investigate the fruits of participatory mental health research, although there is also widespread acknowledgement of how difficult measuring the impact of such research can be.^19^ This echoes the larger move towards measuring impact, where many have expressed concerns with regard to the difficulty of evaluating participatory research, but the majority believe it is important to try to do so.^20^

While the investigation of impact is a worthwhile one, those engaged in this evaluative project tend to focus overwhelmingly on the *epistemic* benefits that can result from involving patients or the public in research, leaving other considerations in the dark. As Kristina Staley and Duncan Barron have aptly pointed out, it is unsurprising that the medical researchers being asked to engage in participatory research, who are steeped in a culture of empirical assessment, tend ‘to think about involvement as an intervention, to seek to evaluate its impact in the same way that treatments are tested, to highlight the need for an evidence base for involvement and to use the language of research to describe its practice and report its outcomes’.^21^, ^22^ This indicates that a focus on epistemic impact may be somewhat inevitable, but as we explain later, its harms, particularly in a realm as complex as psychiatry, are rarely recognized.

## 3 JUSTIFICATIONS FOR INVOLVEMENT IN PSYCHIATRY

### 3.1 Two approaches

The narrow focus of impact in evaluations of participatory research feels especially jarring within the realm of psychiatry, where the involvement of those with lived experience in research is not seen by many as merely an epistemic tool. In contrast, discussions of the justifications of participatory research in psychiatry have long included widespread acknowledgement of the importance of both ethical and epistemic reasons to engage in such research. Diana Rose characterizes two discourses at play, one concerned with ethical reasons for involvement and another concerned with epistemic reasons for involvement:

First, there is a strong current of opinion that says that the type of knowledge generated is not especially relevant because what matters is the *ethical dimension* in that those who research is for should have a stake in how it proceeds. In the second, which has not really been developed at all, it is argued that the value of PPI [public and patient involvement] must be related to how changing the knowledge producers *changes the knowledge.* 7

Peter Beresford also describes a divide within discussions of why of service users ought to be involved in mental health research, representing what he calls a consumerist approach and a democratic approach.^11^ The consumerist approach, Beresford suggests, is conservative and top‐down, interested in the interplay of profits and effectiveness, and achieving instrumental goals through the inclusion of service users. The democratic approach, on the other hand, is explicitly political, closely aligned with the disability rights movement, and committed to ‘inclusion, autonomy, and independence, and the achievement of their human and civil rights’.^11^ Similarly, David Pilgrim distinguishes between survivors and consumers, which map onto whether the perspective of a service user is operating as an expression of protest or as a management resource.^23^ Drawing on these distinctions, Tehseen Noorani describes the differences between spaces dominated by service users, which are ‘used to measure and evaluate service provision’, and survivors/activists, which are used to ‘promote user‐led service development, often reframing the problems faced by mental health‐care governance and the mainstream solutions to these problems’.^24^

### 3.2 Epistemic grounds for involvement

These two approaches represent two different justifications underlying the shift towards service user involvement in mental health research. The first is an epistemic justification, grounded in what is often called expertize through experience. Those thinking about the epistemic reasons why service users ought to be involved in mental health research tend to offer evidence for involvement that aligns with impact assessments and demonstrates the epistemic improvements in research that can come about through participatory research. These improvements are sometimes cashed out as the three R's—rigour, relevance and reach.^25^ Evidence suggests that participatory research often contributes to each of these aims. In terms of rigour, Steve Gillard et al describe how when mental health service users were involved in qualitative analysis of transcripts of interviews about support for self‐care in the UK, their participation led to the identification of novel themes within the data as well as critical reflection on the process of analysis.^18^, ^19^ Research also suggests that interviewees may be more forthcoming about their concerns with mental health services when interviewed by service users.# 8 Relevance is perhaps the most well demonstrated, with countless examples of how participatory research can strengthen the proximity of research methodology to the goals of patients, through the identification of outcome measures that are meaningful to participants, through making a clinical trial accessible to those with a particular condition or by identifying novel research questions that reflect the values of patients.^13^, ^15^, ^17^ The impact of service user involvement on reach is also well documented, as enrolment and retention have both been shown to be positively impacted by patient involvement.|| 26

### 3.3 Ethical grounds for involvement

The second is a moral justification, connected to the idea that those being directly impacted by psychiatric research have a claim to be able to contribute to such research. Those thinking about the ethics of why involvement matters tend to align with the disability rights movement, emphasizing the importance of constructing mental health research that involves ‘nothing about us without us’. Participation in research, in this context, is seen as a claim of those who have been on the receiving side of the mental health system and not as an instrumental tool for researchers. The ethical foundations for participation have their roots in decades of protest and activism of service users, growing out of the distinct but overlapping discourses of mad pride, antipsychiatry, c/s/x groups, the recovery movement and mad studies. While not always united under common critiques and values, each of these movements has recognized the role that power structures play in the field of psychiatry and the damage that can be done when one group is left powerless and deemed to lack the capacity to speak for themselves. Some have focused on human rights abuses that litter the history of psychiatry, from Nazi research to an endless list of ‘treatments’ that tended to do more harm than good.^27^ Others have looked to ways in which psychiatry has been utilized as a tool of oppression, through diagnosing and locking up political dissidents or through including drapetomania and homosexuality in the DSM.^28^, ^29^ Still others have pushed back against the common use of coercion, restraints and forced treatment within the field, resisting the justifications offered for paternalism and identifying the harms that can result from such experiences.^30^, ^31^ Looking to such harms within psychiatry paints a radically different justificatory picture of the field than does looking to epistemic reasons. On one view, participation emerges out of this history as a necessary balance to these wrongs, perhaps as a form of reparation. These focal points differ substantially from those embraced on the epistemic side, offering intrinsic reasons for involvement, as opposed to instrumental ones.

Perhaps unsurprisingly, emphases on these different justifications tend to vary across different stakeholders involved in participatory research. Interviews conducted by Deborah Rutter et al revealed significant distance across stakeholder views of why involvement was taking place in the planning and delivery of mental health services in Trusts in London. Managers of Trusts tended to view involvement as a way to improve services as well as to justify the decisions they made, while nurses saw participation as ‘misplaced political correctness’.^10^ Service users identified a range of justifications for why they were there: ‘empowerment of oppressed group; increased self‐esteem and respect for mental health patients; promotion of citizenship and civil rights; and countering stigma, social exclusion and coercive controls’ as well as to change services and address issues identified by service users.^10^

### 3.4 Links

While epistemic and ethical justifications for participatory psychiatric research can be disentangled, they are also intimately tied to each other. In line with this, Noorani unpacks the difference between service users and survivor/ activists, but also acknowledges their common ground:

What unites both the consumerist subject position of service user and the oppositional subject position of survivor, is that they are granted authority by virtue of their experiences of mental distress and/or of service (ab)use, and seek progressive developments in the treatment, perception and governance of the experiences of mental distress.^24^

However, beyond the shared interests of these two approaches to mental health service users, there is additional reason to think that the epistemic and ethical justifications underlying participatory research cannot come apart cleanly. Within psychiatry, the importance of subjective reports for determining whether an individual is well or not suggests that service user voices are central to research, whether they are treated as contributors or not. One cannot easily conduct research in psychiatry that bypasses the experiences of participants, as one could in a study that involved only objective measures (eg blood cell count). This makes the role of patients particularly central for psychiatric research: their experience directly informs the measurement of the phenomena of interest in the field. This unique epistemic claim in relation to their own illness or wellness suggests that service users in psychiatry may have an ethical claim on participating in the research process. In the other direction, the ethical grounding for participatory research in psychiatry links to the expertize that service users develop through their own experiences of mental distress and through their interactions with the mental health system. If the right of service users to participate and have a say within knowledge production in psychiatry is in part derived from the lack of opportunities, agency and power they have found within the mental health system, these denials are also likely to contribute to a unique perspective of the system, which can help make research more relevant and impactful. The bidirectional relationships between epistemic and ethical justifications underlying service user involvement in mental health research indicate that, as Su has suggested, the ‘instrumental and normative concerns are irrevocably interwoven’ within participatory research.^12^

## 4 HARMS OF A FOCUS ON IMPACT

Whether or not they can be untangled, both epistemic and ethical reasons underlie the process of democratizing the production of knowledge within psychiatry. Service users have not fought for a voice at the table merely to help improve the research process, but because they have a right to be there. This suggests that what justifies involvement is much larger than that captured by the epistemic focus of those seeking to evaluate the impact of participatory research. Furthermore, this raises a worry about the potential of the push towards measuring the instrumental impact of participatory research: by narrowing in on merely the epistemic benefits of involvement, the ethical reasons can become obscured.

### 4.1 Skepticism

As the quest to build an evidence base that is systematic and quantitative continues, participatory health research comes to be seen through a single lens.^32^ If the positive impact of such research is borne out in the data, the results are used to convince doubtful researchers that participatory research is worthwhile because it will make their research better. This contributes to the view among researchers that the point of involving those impacted by their research in the process is merely to help them achieve their research goals. On the other hand, if the improvements that they anticipate are not borne out by the research, investigators will understandably be disappointed and may become (more) skeptical about the importance of participatory research. This skepticism could serve to exacerbate well‐documented, existing difficulties in participatory research related to role confusion and tokenism.^9^, ^13^

### 4.2 Tokenism

When investigators are required to involve patients or the public in their research, but are unsure of how to do so or are doubtful about what they might have to contribute, involving them in a tokenistic, non‐committal way is common.^9^, ^16^ One researcher described their teams’ experience: ‘It was a degree of tokenism and I say that completely openly… actually none of us knew quite why or what the patient's role would be or how it would work out’.^33^ Because participatory research done without a clear aim or understanding of the process rarely leads to significant benefits, such instances serve to ‘reinforce status quo assumptions about the inability of community members to “speak” to researchers and policymakers, or to directly contribute to the “scientific” literature’.^34^ This highlights what has been called the self‐fulfilling prophecy of tokenism in participatory research: ‘PI [public involvement] when undervalued leads to tokenism in involvement practice; tokenistic practice fails to demonstrate the value of PI; and hence, PI is therefore perceived as not adding value to health and social care research’.^35^

In mental health research, such tokenism is common.^36^, ^37^ Nev Jones et al acknowledge that in psychiatry, ‘far too often, co‐researchers or community members do not in fact have meaningful influence over the production and dissemination of knowledge’,^34^ while Beresford notes that ‘where user or “consumer” involvement is required by research funders, it is frequently treated as a “box‐ticking” exercise and seen by some researchers more as a nuisance than of any real importance’.^11^ In a case study of participation of service users, carers and public housing tenants in policy developments surrounding housing for people with psychiatric disabilities, Sam Battams and Anne Johnson describe how ‘the consumer groups that did exist were largely an “end” in themselves rather than a “means” to more empowering forms of participation’.^38^

### 4.3 Negative experiences

Furthermore, participatory research, when done badly, has the potential to exacerbate harm to service users, through disempowering or stigmatizing experiences.^36^ Lydia Lewis recounts an interview with a service user who felt undermined by others in meetings who would dismiss her views on the basis of her diagnosis or symptoms: ‘There's always, when you say something controversial, “oh well she's not feeling very well at the moment” muttered under people's breath’.^39^ This took place within a context in which service users were being asked to inform the development and planning of mental health services in the north‐east of Scotland, a policy initiative meant to ‘ensure recognition of service users and their (potential) contributions to service delivery’.^39^ Participatory efforts can be undermined by stereotypes or patterns of epistemic injustice, and stigmatizing encounters can occur amongst service users as well as between service users and others.^40^ A focus on the impact of participation on research fails to recognize how, even if research is improved through participatory methods, those involved can be harmed by the process.^16^

## 5 MOVING BEYOND THE EPISTEMIC

### 5.1 Looking at power

As demonstrated above, a focus on purely the epistemic or instrumental reasons to engage in participatory research in psychiatry risks limiting conversations to only half the story. In order to preserve the crucial ethical reasons why involvement matters within the sphere of mental health research, the focus needs to be not only on the impact of participation in research, but the way in which power is distributed in knowledge making projects within psychiatry. As Rose has described it, ‘one of the founding principles of participatory research.. is that it should level the power relations between researchers and the community in the research itself’.^41^ This responds to the ethical justifications for participatory research more than the epistemic ones, suggesting that sharing power will be central to an appropriate response to the ethical reasons for involvement. In a related discussion of how mental health research could be transformed through service user involvment, Jones et al, drawing on Foucault, offer this description of power:

[An] individual does not ‘possess’ power as she would a material good, but rather exercises power in the context of a net of social and institutional relations that variably reinforce, challenge, potentiate, or otherwise structure her actions. Thus, a researcher’s decision to do, for example, X or Y, would not be seen as simply ‘her’ decision but rather as an act that (a) has meaning and influence because of a variety of contextual social, political, and institutional factors and (b) in its own right reinforces, challenges, or (re)structures social and political discourse.^34^

This description of power points to the broad distribution and many variables that contribute to power within a research enterprise, suggesting that a shift in power dynamics will require more than a few consultations with service users within research. Here, we offer some preliminary thoughts on what implications fall out of recognizing the ethical reasons for involvement as well as the epistemic ones.

### 5.2 Expanding the evaluation of participatory research

Acknowledging both ethical and epistemic reasons for involvement raises the question of how to measure successful participatory mental health research so that both kinds of reasons for participation are taken up in evaluation. One might suggest that merely increasing the amount of involvement taking place is the answer, moving away from impact and measuring quantity. However, as indicated above, participatory research that is done badly can cause harm, by further stigmatizing and disempowering those involved, even if it ‘improves’ the research process according to some measures (eg recruitment). Furthermore, more participatory research can mean more participation that fails to be representative. Ginny Russell and colleagues describe a case in which a consortium for autism research sought to involve the voices of those diagnosed with autism and their carers.^42^ The way participation was structured, however, ensured that only those who agreed with the basic premises of the research (eg that autism is best thought of as an illness to be treated, that medications are a suitable treatment) were included in the process. They labelled this ‘selective PPI [patient and public involvement]’, in that it selected for those who already agreed with the research being done and were unlikely to cause trouble. This resulted in a case in which ‘PPI guidelines were followed by the biomedical consortium to the letter, but in a way that involved only those community voices that supported the consortium's research agenda’.^42^

Moving past impact means looking at the quality of participatory research in relation to the ethical demands of service users. Most importantly, this requires the difficult task of examining the role that power is playing in existing participatory projects with an eye to power imbalances. Such an examination might involve measuring how many instances of survivor research and co‐production are taking place as well as how effectively power is being shared within these projects. It might also involve examining involvement in the early phases of research and comparing the existence of projects driven by patient or community needs versus top‐down academic projects. While some have sought to capture the views of service users, researchers and communities engaged in participatory research in impact assessments, more can be done to examine how power is distributed across different participatory projects.^16^ Importantly, power need not be shared entirely with service users in every research project. In some cases, there may be good ethical and epistemic reasons for participation to be minimal (eg an early stage neuroscientific project), while in other cases, it may be imperative that service users are centrally involved from the start or leading the research (eg a project examining the role of coercion in inpatient units).

Furthermore, questions of who power is being shared with are central to an evaluation of participatory research that is responsive to ethical reasons. Many have pointed to issues of representation within participatory mental health research, particularly in terms of the failure to involve racial or ethnic minorities.^43^ Given the diversity of views of service users in relation to mental health, and the way in which diagnosis and treatments impact different populations differently (eg along lines of socioeconomic status, race), this is especially important in the domain of psychiatry.

### 5.3 Developing service user capacities

A second implication of taking into account both the ethical and epistemic reasons for the participatory turn in psychiatry is that the development of service user capacities should be prioritized. If power is going to be truly, not merely tokenisticly, shared between those on either side of the research divide, all parties must have the tools, skills and experience to contribute to such research. This means capacity building in not only qualitative research, where significant strides have been made, but also in quantitative research and clinical trials, where involvement is less common.^34^ The responsibilities for developing service user capacities cannot be taken up by one group of stakeholders alone, but will fall on researchers, funders, institutions, journals and service user communities; as such, it will be no easy task. Recognizing the importance of developing service user capacities in mental health research pushes against the common concern that participatory research should not involve patients who have become ‘professionalized’.^9^ This concern stems from worries that patients with a long history of participatory experience are no longer likely to be representative of the population they are meant to speak for, or are unlikely to be critical of or speak up about issues, because they depend on further involvement for their livelihood.

While these worries are real, the risks of failing to integrate those with lived experience into the upper ranks of research are greater. If service users are not supported in gaining skills to lead and contribute substantially to research projects, the hierarchy between those doing the research and those who are the subjects of research is maintained. Furthermore, when participatory research only involves individuals who are able to contribute to some aspects of the research process, authority over other aspects of the process will always rest in the hands of the researchers, limiting the participatory nature of the research. It should be acknowledged, however, that developing research skills and capacities within communities of patients risks bringing about an ‘increasingly stratified service user movement and rearticulating the hierarchies of psychiatry’.^44^ Issues of representation must therefore also be central to initiatives that seek to build capacities within service users.

### 5.4 Sharing power in different ways

Another implication of looking beyond the epistemic benefits of participatory research and towards the ethical reasons that underlie such research is that power‐sharing need not be restricted to the research process. Involvement of service users in policymaking, funding allocation and service delivery related to mental health can also help to reshape power structures, responding not only to epistemic, but to ethical reasons to reshape the discipline of psychiatry. Furthermore, given that ethical reasons to share authority in the research process stem from long‐standing abuse in research and practice, and from the discipline's ties to political and social oppression, other models of repairing past harms with communities suggest themselves. The development of tribal IRBs, in which indigenous people are given authority over the ethical oversight of research occurring within their communities, has helped to prevent harmful research from being replicated and has allowed for communities to build novel standards of research ethics that are responsive to the unique history and challenges that arise in the postcolonial context.^45^ These novel standards require cultural sensitivity, restore trust in communities that have had negative experiences with research, shift power and data ownership into the hands of those being researched and require attention to community‐level concerns.^46^ Taking note from these developments, other vulnerable research populations have formed grassroots community review boards, in order to help motivate researchers to move towards more appropriate models of research in their communities as well.^47^ This suggests that rethinking representation in research ethics oversight within the domain of psychiatry might be a worthwhile route to sharing power and changing research norms within the discipline.

### 5.5 Conclusion

The participatory turn within the health sciences has brought with it significant developments in terms of involving patients and publics in the research process. It has been followed, however, with a push for measuring impact assessments, in order to determine precisely what changes to research occur as a result of participation. In a field like psychiatry, the focus on impact has the potential to obscure ethical reasons for involvement in favour of merely epistemic ones. Those who have fought for a chance to have a voice at the table and contribute to knowledge about themselves have long emphasized the ties between psychiatry and oppression, the history of abuse within both research and practice, and the way in which service users have been stripped of their agency and subjected to coercive treatments in the past. Considering these aspects of the field as justifications for participatory psychiatry points away from looking merely at impact and towards an investigation of power structures within the discipline. We suggest that, as a start, taking ethical reasons for involvement seriously will mean measuring more than impact, building service user capacities and sharing power in other domains.

## CONFLICTS OF INTEREST

We have no conflicts of interest to report.

## Footnotes

* This is especially true in the United Kingdom, where government bodies have enthusiastically taken up the participatory turn.^1^, ^2^

† PAR, which stems from a more radical history than many of the new forms of participatory research, now has several strains, including community‐based participatory action research and critical participatory action research.^3^

‡ We use the term participatory research within this manuscript to refer to ‘research being carried out “with” or “by”’ those likely to be impacted by the research ‘rather than “to”, “about” or “for” them’, focusing on projects that aim to produce generalizable knowledge and excluding activities geared towards public engagement or education.^4^ While the terms ‘public’ and ‘patient’ are often run together within this literature, there are important differences between the justifications for involving either population.^5^

§ The involvement of carers is also an important dimension of participatory research that is distinct from the involvement of the public, given the expertize that comes from caring from someone who is unwell. While it is often grouped together with the involvement of patients, much of our analysis in this manuscript is specific to the experiences of service users in psychiatry and so cannot be easily extended to carers as well. For a helpful categorization of involvement of the public, laypersons, citizens, patients, carers and advocates, see.^6^

¶ Most impact assessments, understandably, focus on change. As Joanna Crocker et al have pointed out, however, having an impact on research does not always mean changing the research; sometimes, it just means validating an existing idea.^17^

# Although as Emma Simpson and Allan House point out, these findings ‘might be due to clients feeling more able to be honest with users, thus increasing validity, or it might be that they perceive dissatisfaction as the socially desirable response’.^8^

|| This is especially the case when those being involved are patients with relevant experience/ expertize of the condition being studied. See^26^ for more discussion.
